# Development of a short-term prognostic model for anti-N-methyl-D-aspartate receptor encephalitis in Chinese patients

**DOI:** 10.1186/s12883-024-03724-x

**Published:** 2024-08-09

**Authors:** Jingxiao Zhang, Yatong Li, Lei Liu, Feifei Dai, Yujing Peng, Qiuying Ma, Lin Li, Yu Hong, Aihua Liu, Xinghu Zhang, Xiaohui Wang, Junying He, Hui Bu, Yanjun Guo, Hanqiu Jiang, Shilei Cui, Houliang Sun, Jiawei Wang

**Affiliations:** 1 Department of Neurology, Beijing Tongren Hospital, Capital Medical University, Beijing, China; 2 Department of Neurology, Xuanwu Hospital, Capital Medical University, Beijing, China; 3 Department of Neurology, Beijing Tiantan Hospital, Capital Medical University, Beijing, China; 4 Beijing Children Hospital, Capital Medical University, Beijing, China; 5 Department of Neurology, the Second Hospital of Hebei Medical University, Shijiazhuang, China

**Keywords:** anti-N-methyl-D-aspartate receptor encephalitis, Immunotherapy, Prediction, Prognosis, Nomogram

## Abstract

**Background:**

Recognizing the predictors of poor short-term prognosis after first-line immunotherapy in patients with anti-N-methyl-D-aspartate receptor (anti-NMDAR) encephalitis is essential for individualized treatment strategy. The objective of this study was to ascertain the factors that forecast short-term prognosis in patients with anti-NMDAR encephalitis, develop a prognostic prediction model, and authenticate its efficacy in an external validation cohort. Further, all patients were followed-up long-term to assess the factors of long-term outcome and relapses.

**Methods:**

A prospective enrollment of patients diagnosed with anti-NMDAR encephalitis was conducted across five clinical centers in China from June 2014 to Mar 2022. The enrolled patients were divided into the derivation and validation sets based on enrollment time. The short-term prognostic model was visualized using a nomogram. Further, all patients were followed-up long-term to assess the factors of long-term outcome.

**Results:**

This study found that poor short-term prognosis was a risk factor for poor long-term outcome (6-month prognosis, OR 29.792, 95%CI 6.507-136.398, *p* < 0.001; 12-month prognosis, OR 15.756, 95%CI 3.384–73.075, *p* < 0.001; 24-month prognosis, OR 5.500, 95%CI 1.045–28.955, *p* = 0.044). Abnormal behavior or cognitive dysfunction (OR 8.57, 95%CI 1.48–49.79, *p* = 0.017), consciousness impairment (OR19.32, 95%CI 3.03-123.09, *p* = 0.002), autonomic dysfunction or central hypoventilation (OR 5.66, 95%CI 1.25–25.75, *p* = 0.025), CSF pleocytosis (OR 4.33, 95%CI 1.48–12.65, *p* = 0.007), abnormal EEG (OR 5.48, 95% CI 1.09–27.54, *p* = 0.039) were independent predictors for a poor short-term prognosis after first-line immunotherapy. A nomogram that incorporated those factors showed good discrimination and calibration abilities. The area under the curve (AUC) for the prognostic model were 0.866 (95%CI: 0.798–0.934) with a sensitivity of 0.761 and specificity of 0.869.

**Conclusion:**

We established and validated a prognostic model that can provide individual prediction of short-term prognosis after first-line immunotherapy for patients with anti-NMDAR encephalitis. This practical prognostic model may help neurologists to predict the short-term prognosis early and potentially assist in adjusting appropriate treatment timely.

**Supplementary Information:**

The online version contains supplementary material available at 10.1186/s12883-024-03724-x.

## Background

Anti-N-methyl-D-aspartate receptor (anti-NMDAR) encephalitis is the most common type of autoimmune encephalitis (AE), with a wide spectrum of symptoms that includes acute or subacute abnormal behavior, cognitive dysfunction, epilepsy, consciousness impairment, and autonomic dysfunction [1, 2]. Although symptoms can be severe and potentially life-threatening in the acute phase, fortunately, it is still a treatable disease.

Current treatment strategies for anti-NMDAR encephalitis are mostly based on immunotherapy. First-line immunotherapy (corticosteroids, intravenous immunoglobulin and plasma exchange/immunoadsorption) and second-line immunotherapy (rituximab, cyclophosphamide, azathioprine and mycophenolate mofetil) are the mainstay of treatment [3, 4]. In addition to conventional immunotherapy, interleukin (IL)-6 inhibition (tocilizumab), anti-CD38 monoclonal antibody (daratumumab), and proteasome inhibitors that block plasma-cell generation (bortezomib) may be beneficial for those who do not respond quickly to conventional immunotherapy [5–7]. Early aggressive immunotherapy is associated with good outcomes [8–10].

Although anti-NMDAR encephalitis is treatable, approximately half of patients respond poorly to first-line immunotherapy when assess at discharge [11–13]. Adding second-line immunotherapy is usually beneficial when first-line treatments failed [11, 13]. The course of the disease and the response to treatment fluctuate from patient to patient, thus it is difficult to predict the response to treatment at the disease onset. However, understanding what factors may influence the response to treatment and predicting the prognosis early are essential in adjusting treatment timely. Integration of various prognostic factors into a nomogram is a visualization tool for neurologists to predict the short-term prognosis after first-line immunotherapy in anti-NMDAR encephalitis, which may be helpful to adjust appropriate treatment timely and significantly improve the outcomes. However, nomograms to predict short-term prognosis in anti-NMDA encephalitis have not been fully characterized. To date, several studies have been focused on predictors associated with prognosis and prognostic models have been constructed [11, 14–18]. However, some of them were based on all types of AE, the heterogeneity of AE may lead to inaccuracy when using these factors to assess anti-NMDAR encephalitis. Some of them were limited to small sample size. Little is known about the factors affecting the response to first-line immunotherapy in patients with anti-NMDAR encephalitis and it is difficult to predict the short-term prognosis.

In this study, we aimed to further investigate the factors affecting short-term prognosis after first-line immunotherapy in patients with anti-NMDAR encephalitis based on the data from a multicenter registry in China, and to develop a short-term prognostic model by incorporating known clinical variables to predict the prognosis. In addition, we used a separate cohort to externally validate it. Furthermore, we had a long-term follow up with all patients to investigate the predictors of long-term prognosis.

## Methods

### Study design and participants

Data used in this study were obtained from a study named The Multicenter and Prospective Clinical Registry Study of Anti-NMDAR Encephalitis in Beijing Area (Clinicaltrials.gov number: NCT02443350), which was a multicenter prospective clinically registered study with consecutive suspected patients with encephalitis conducted at five clinical centers in China. We enrolled patients with anti-NMDAR encephalitis between June 10, 2014, and March 20, 2022. The enrolled patients met the following included criteria: (1) patients with a diagnosis of definite anti-NMDAR encephalitis according to Graus and Dalmau criteria [2]. (2) received first-line immunotherapy, including corticosteroids, intravenous immunoglobulins (IVIG), or plasma exchange/immunoadsorption in the acute phase. The excluded criteria included: (1) patients whose serum and cerebrospinal fluid (CSF) were both negative for antineuronal antibodies; (2) other types of AE; (3) patients who received second-line immunotherapy prior to this clinical attack; (4) incomplete clinical data. Among the included patients, those who enrolled between June, 10, 2014 and December, 31, 2017 were used for the derivation cohort, and those who enrolled between January, 1, 2018 and March, 20, 2022 were used for the external validation cohort. To develop a robust model, at least 10 events for each predictor parameter were acquired [19]. Furthermore, Follow-up was conducted regularly for all patients enrolled to investigate the predictors of long-term prognosis. This study was reviewed and approved by the Ethics Committee of Beijing Tongren Hospital, Capital Medical University (approval code: BJFH-EC/2013-024).

### Clinical assessment and prognosis evaluation

All patients underwent neurologic examination including CSF analysis, cranial magnetic resonance imaging (MRI) and electroencephalogram (EEG) in the acute phase. The presence of anti-NMDAR antibodies in serum or CSF was tested with a cell-based assay. To screen for tumors, all patients underwent CT scans of the thorax, abdomen and pelvis, or B ultrasound of the abdomen and pelvis, or positron emission tomography-computed tomography scan when necessary. All patients received first-line immunotherapy including corticosteroids, IVIG, plasma exchange/immunoadsorption, alone or combined. Neurological status was assessed with the modified Rankin scale (mRS) within four weeks after finishing one round of first-line immunotherapy, and if any second-line immunotherapy was used, the neurological status was assessed prior to the start of second-line immunotherapy. Follow-up was conducted in all patients by outpatient review or telephone follow-up which were completed twice in the first and sixth month and once annually thereafter.

### Definition of variables

Abnormal MRI was defined as hyperintense signal on T2-weighted fluid-attenuated inversion recovery sequences highly restricted to one or both medial temporal lobes (limbic encephalitis), or in multifocal areas involving grey matter, white matter, or both, compatible with demyelination or inflammation [2]. Abnormal EEG included the presence of any of the following: abnormal state changes, focal or diffuse slowing, epileptiform discharges, rhythmic slowing, extreme delta brush, or electrographic seizures [14]. Early immunotherapy was defined as initiation of immunotherapy within four weeks from disease onset [14]. Patients were divided into two groups according to mRS score assessed after first-line immunotherapy. Patients with an mRS ≤ 2 were considered to have a “good prognosis”, representing a continuum of function without disability (mRS 0) to slight disability yet independent living (mRS 2). In contrast, patients with an mRS > 2 were considered to have a “poor prognosis”, which ranged from moderate disability requiring assistance for completely independent living but being able to walk independently (mRS 3), to severe disability, being bed and requiring continuous nursing care (mRS 5) and death (mRS 6). The mid-to-long term prognosis was defined as the prognosis assessed at the 6-month mark. The long-term prognosis was defined as the prognosis evaluated at a follow-up time equal to or exceeding 12 months.

### Date collection

The neurological status and following factors were analyzed: (1) demographics, including sex and age; (2) prodromal symptoms, including fever, headache, respiratory symptoms, vomiting, diarrhea; (3) six major groups of clinical symptoms, including abnormal behavior or cognitive dysfunction, speech dysfunction, seizures, movement disorder, consciousness impairment, autonomic dysfunction or central hypoventilation; (4) auxiliary examination findings, including CSF white blood cell count, CSF protein, cranial MRI findings, EEG findings; (5) accompanied tumor or tumor history; (6) the interval from disease onset to the initiation of immunotherapy; (7) short-term prognosis after first-line immunotherapy; (8) long-term prognosis.

### Statistic analysis

Continuous variables were presented as medians and ranges for nonnormal distribution. Categorical variables were expressed as frequencies and percentages. The Mann–Whitney U test was used for intergroup comparisons of continuous variables and the *chi-square* or *Fisher’s* exact test was used for intergroup comparisons of categorical variables. The candidate variables with a univariate relation (*p* < 0.1) with a poor prognosis were included in the multivariate logistic regression by a backward step-down selection process to determine the independent predictors of a poor prognosis. Odds ratio (OR) and 95% confidence intervals (95%CI) were obtained. The *p*-value < 0.05 was considered of statistical significance. Statistical analyses to identify independent prognostic factors were conducted in IBM SPSS 26.0.

The nomogram and forest plot were formulated by R 4.2.1 based on the results of the multivariable analysis. The performance of the nomogram to predict a poor short-term prognosis included its discrimination and calibration. The discrimination was measured by the area under the curve (AUC) of receiver-operator characteristic curve (ROC). The calibration was assessed by the calibration curves. The nomogram was subjected to 1000 bootstrap resamples for internal validation and externally validated with another validation cohort.

## Result

### Patients characteristics in the derivation and validation cohorts

**Fig. 1:**
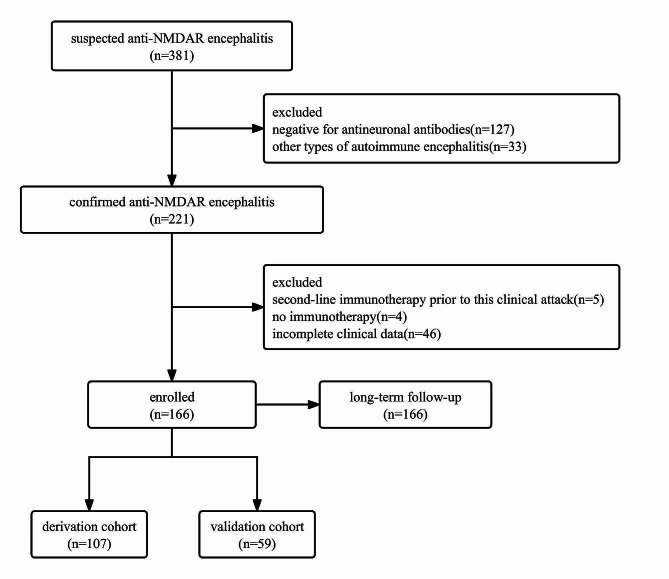
The flowchart of patient enrollment. 381 suspected anti-NMDAR encephalitis patients were collected, 166 patients were eventually included after exclusion of ineligible patients. Among them, 107 patients admitted between June 10, 2014 and December 31, 2017 were used for the derivation cohort, another 59 patients admitted between January 1, 2018 and March 20, 2022 were used for the external validation cohort. Long-term Follow-up was conducted regularly for all patients enrolled

A total of 381 suspected anti-NMDAR encephalitis patients were collected. After exclusion of ineligible patients, 166 patients were eventually included. The included patients were divided into derivation cohorts and validation cohorts according to their admission time, 107 patients admitted between June, 10, 2014 and December, 31, 2017 were used for the derivation cohort, another 59 patients admitted between January, 1, 2018 and March, 20, 2022 were used for the external validation cohort. No statistically significant differences in the baseline demographics and clinical variables were found between the two cohorts (*p* > 0.05), except for age (*p* < 0.001), speech dysfunction (*p* < 0.001), and movement disorder (*p* < 0.001). The demographics and clinical characteristics of patients in derivation and validation cohorts were displayed in Table 1. Long-term follow-up of all patients was also performed to explore the factors influencing the long-term prognosis of anti-NMDAR encephalitis (Fig. 1).

**Table 1:** The demographics and clinical characteristics of anti-NMDAR encephalitis in the training and validation cohorts

Characteristics	Derivation cohort (*n* = 107)	Validation cohort (*n* = 59)	*p*-value
Gender, Male	47 (43.93)	29 (49.15)	0.518
Age	13 (2–72)	26 (2–78)	< 0.001
Prodromal symptoms	40 (37.38)	21 (35.59)	0.819
Clinical manifestations			
Abnormal behavior or cognitive dysfunction	87 (81.31)	45 (76.27)	0.441
Speech dysfunction	49 (45.79)	5 (8.47)	< 0.001
Seizures	83 (77.57)	42 (71.19)	0.361
Movement disorder	64 (59.81)	14 (23.73)	< 0.001
Consciousness impairment	19 (17.76)	7 (11.86)	0.393
Autonomic dysfunction or central hypoventilation	18 (16.82)	7 (11.86)	0.393
auxiliary examination			
CSF pleocytosis	38 (35.51)	30 (50.85)	0.054
Elevated CSF protein	21 (19.63)	16 (27.12)	0.267
Abnormal MRI	47 (43.93)	24 (40.68)	0.686
Abnormal EEG	85(79.44)	52 (88.14)	0.158
Tumor	4 (3.74)	6 (10.17)	0.096
Early immunotherapy	82 (76.64)	40 (67.80)	0.273
Poor prognosis	46 (42.99)	25 (42.37)	0.939

CSF: Cerebrospinal fluid; EEG: electroencephalogram; MRI: magnetic resonance imaging.

### Independent prognostic factors in the derivation cohort

Univariate analysis indicated that consciousness impairment (*p* < 0.001), autonomic dysfunction or central hypoventilation (*p* < 0.001), CSF pleocytosis (*p* = 0.002), abnormal EEG (*p* < 0.001) may be associated with a poor short-term prognosis (Table 2). All analyzed variables with a *p* < 0.1 in univariate analysis were entered into the multivariate analysis. The multivariate logistic regression analysis indicated that abnormal behavior or cognitive dysfunction (OR 8.57, 95%CI 1.48–49.79, *p* = 0.017), consciousness impairment (OR19.32, 95%CI 3.03-123.09, *p* = 0.002), autonomic dysfunction or central hypoventilation (OR 5.66, 95%CI 1.25–25.75, *p* = 0.025), CSF pleocytosis (OR 4.33, 95%CI 1.48–12.65, *p* = 0.007), abnormal EEG (OR 5.48, 95% CI 1.09–27.54, *p* = 0.039) were still associated with a poor short-term prognosis, those variables were included in the final multivariable prediction model (Table 3).

**Table 2:** Results of univariate binary logistic regression analysis of variables and prognosis in the derivation cohort

Variable	Good prognosis (*n* = 61)	Poor prognosis (*n* = 46)	*p*-value
Gender, Male	29 (47.54)	18 (39.13)	0.385
Age	13 (3–64)	12.5 (2–72)	0.738
Prodromal symptoms	22 (36.07)	18 (39.13)	0.746
Abnormal behavior or cognitive dysfunction	46 (75.41)	41(89.13)	0.071
Speech dysfunction	27 (44.26)	22 (47.82)	0.714
Seizures	46 (75.41)	37 (80.43)	0.537
Movement disorder	36 (59.16)	28 (60.87)	0.847
Consciousness impairment	2 (3.28)	17 (36.96)	< 0.001
Autonomic dysfunction or central hypoventilation	3 (4.92)	15 (32.61)	< 0.001
CSF pleocytosis	14 (22.95)	24 (52.17)	0.002
Elevated CSF protein	13 (21.31)	8 (17.39)	0.613
Abnormal MRI	23 (37.70)	24 (52.17)	0.135
Abnormal EEG	41 (67.21)	44 (95.65)	< 0.001
Tumor	2 (3.28)	2 (4.35)	1.000
Early immunotherapy	44 (72.13)	37 (80.43)	0.321

CSF: Cerebrospinal fluid; EEG: electroencephalogram; MRI: magnetic resonance imaging. *Data are presented as frequencies (percentages) or medians (ranges), Univariate binary logistic regression analysis was conducted using the chi-square test or Mann-Whitney U-test, the *p*-value was derived from bivariate association analyses between variables and the prognosis

**Table 3:** Variables for poor prognosis in final regression model in the derivation cohort

Variable	OR (95%CI)	*p*-value
Abnormal behavior or cognitive dysfunction	8.57 (1.48–49.79)	0.017
Autonomic dysfunction or central hypoventilation	5.66 (1.25–25.75)	0.025
Consciousness impairment	19.32 (3.03-123.09)	0.002
CSF pleocytosis	4.33 (1.48–12.65)	0.007
Abnormal EEG	5.48 (1.09–27.54)	0.039

CSF: Cerebrospinal fluid; CI: confidence interval; EEG: electroencephalogram; OR: odds ratio

### Prognostic nomogram

**Fig. 2:**
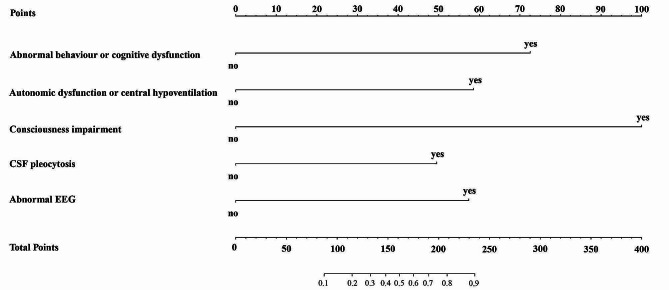
The nomogram to estimate the probability of a poor short-term prognosis after first-line immunotherapy in anti-NMDAR encephalitis. The points of five factors determined on the upper point scale were added to get the total points, draw a vertical line to the last axis to get the corresponding probability of a poor prognosis

A nomogram that incorporated the significant prognostic factors based on the multivariate analysis was established. As shown in Fig. 2, the nomogram illustrated that consciousness impairment contributed the most to prognosis, followed by abnormal behavior or cognitive dysfunction, autonomic dysfunction or central hypoventilation and abnormal EEG. CSF pleocytosis showed a moderate effect on prognosis. Each factor was assigned a point on the upper point scale, the points of five factors were added to get the total points, draw a vertical line to the last axis to get the corresponding probability of a poor short-term prognosis.

### Calibration and validation of the nomogram

**Fig. 3:**
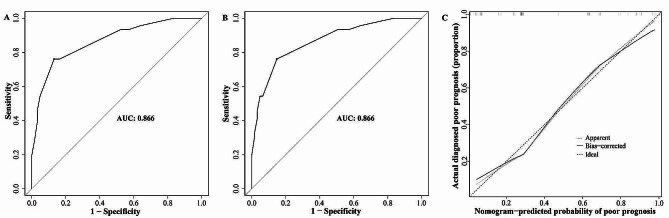
The ROC curve and calibration curve of the nomogram in the derivation cohort. (**A**) The ROC curve of the nomogram, with an AUC of 0.866 with a sensitivity of 0.761 and specificity of 0.869. (**B**) The ROC curve of the nomogram after internal validation using 1000 bootstrap resamples, with an AUC of 0.866 with a sensitivity of 0.761 and specificity of 0.852. (**C**) The calibration curve of the nomogram. Both the apparent calibration curve and the bias-corrected calibration curve after 1000 bootstrap resamples demonstrated good agreement between prediction and observation

**Fig. 4:**
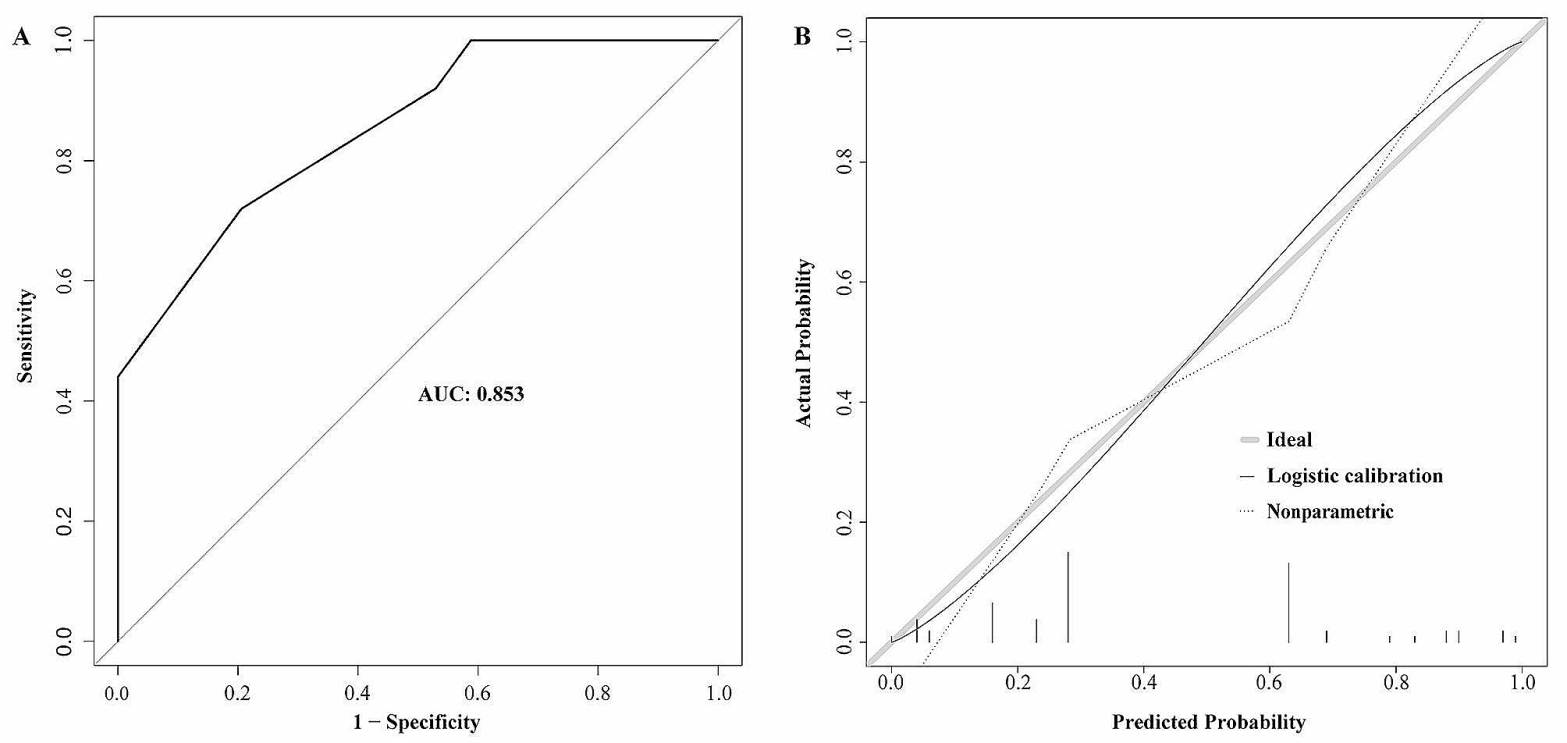
The ROC curve and calibration curve of the nomogram in the validation cohort. (**A**) The ROC curve of the nomogram, with an AUC of 0.853 with a sensitivity of 0.720 and specificity of 0.794. (**B**) The calibration curve of the nomogram. The solid black line represents logistic calibration curve, and the dotted line represents data for the validation cohort

The AUC was calculated to determine the nomogram’s discriminatory capacity. The nomogram demonstrated good discrimination accuracy, with an AUC of 0.866 (95%CI: 0.798–0.934) with a sensitivity of 0.761 and specificity of 0.869 in derivation cohort and 0.866 (95%CI: 0.799–0.934) with a sensitivity of 0.761 and specificity of 0.852 after internal validation using 1000 bootstrap resamples (Fig. 3A and B). The calibration of the nomogram was performed by comparing the predicted probability of poor prognosis with the actual diagnosed poor prognosis after bias correction. The apparent calibration curve, which represented the calibration of the model in the original data set, was close to the ideal line, indicating that the observed probability was consistent with the predicted probability. The bias-corrected calibration curve, which represented the result after correcting with 1000 bootstrap resamples, also demonstrated that the prediction model was well calibrated (Fig. 3C). For the external validation, the AUC was 0.853 (95%CI 0.762–0.944) with a sensitivity of 0.720 and specificity of 0.794 and the calibration curve showed great calibration (Fig. 4).

### Analysis of factors influencing the long-term prognosis of anti-NMDAR encephalitis

**Fig. 5:**
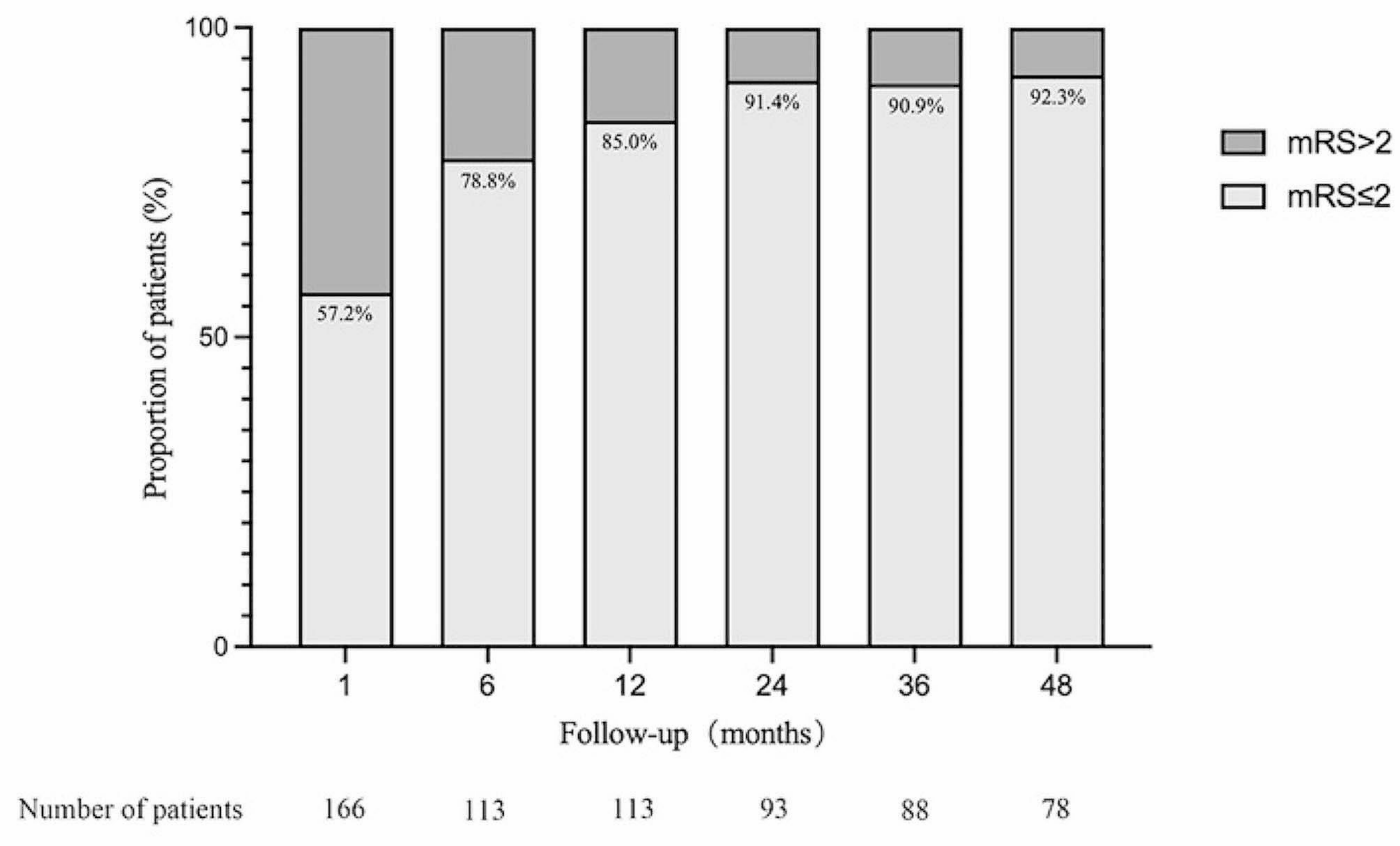
Prognosis of anti-NMDAR encephalitis. The prognosis of patients at months 1, 6, 12, 24, 36, and 48 is demonstrated. The proportions with good prognosis were 57.2%, 78.8%, 85.9%, 91.4%, 90.9% and 92.3%, respectively

Participants were followed up four weeks after completion of first-line immunotherapy, and 95 patients (57.2%) had a good outcome (mRS score ≤ 2). By 6 months, a total of 113 patients were followed up and 89 (78.8%) had a good prognosis. By 12 months, a total of 113 patients were followed up, of whom 96 (85.0%) had a good prognosis. By 24 months, a total of 93 patients were followed up, of whom 85 (91.40%) had a good prognosis. By 36 months, a total of 88 patients were followed up and 80 (90.9%) had a good prognosis. By 48 months, a total of 78 patients were followed up and 72 (92.31%) had a favorable prognosis (Fig. 5). Median follow-up time was 24 months (maximum, 105 months).

To investigate the impact of poor short-term prognosis after first-line immunotherapy on long-term prognosis, short-term prognosis after first-line immunotherapy was included as a variable in logistic regression analyses to further analyze the factors influencing the prognosis of patients with anti-NMDAR encephalitis at 6 months, 12 months, and 24 months. Univariate logistic regression analysis showed that consciousness impairment (OR 13.844, 95%CI 4.546–42.164, *p* < 0.001), autonomic dysfunction or central hypoventilation (OR 7.232, 95%CI 2.434–21.491, *p* < 0.001), and poor short-term prognosis after first-line immunotherapy (OR 29.792 95%CI 6.507-136.398, *p* < 0.001) may be associated with poor prognosis at 6 months. Age (OR 1.036, 95%CI 1.005–1.068, *p* = 0.024), consciousness impairment (OR 12.286, 95%CI 3.825–39.463, *p* < 0.001), autonomic dysfunction or central hypoventilation (OR 5.409, 95%CI 1.709–17.119, *p* = 0.004), and poor short-term prognosis after first-line immunotherapy (OR 15.756, 95%CI 3.384–73.075, *p* < 0.001) may be associated with poor prognosis at 12 months. Age (OR 1.063, 95%CI 1.021–1.107, *p* = 0.003), epilepsy (OR 0.173, 95%CI 0.038–0.789, *p* = 0.024), consciousness impairment (OR 10.139, 95%CI 2.139–48.063, *p* = 0.004), and poor short-term prognosis after first-line immunotherapy (OR5.500, 95%CI 1.045–28.955, *p* = 0.044) may be associated with poor prognosis at 24 months (Supplemental Table 1).

**Fig. 6:**
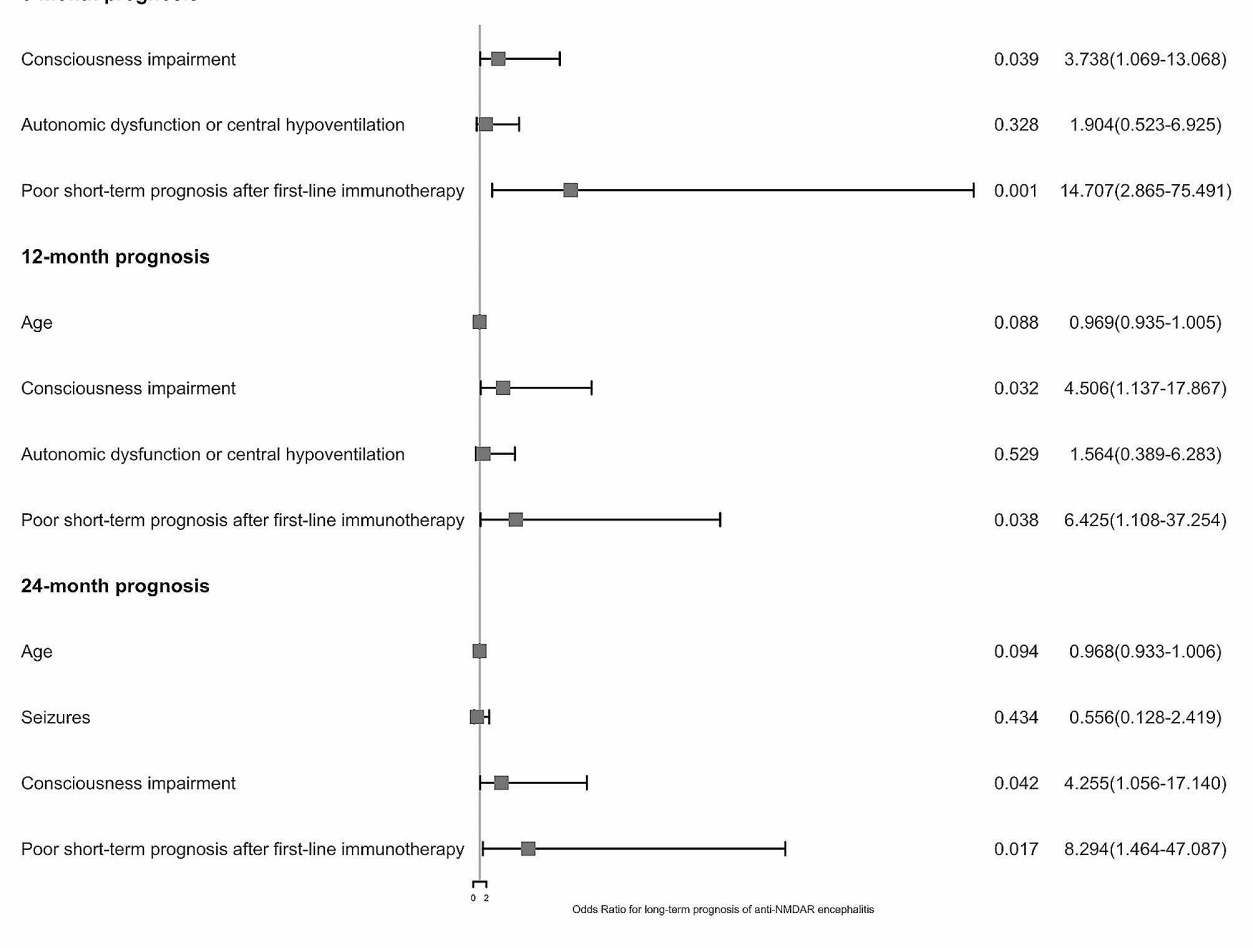
The forest plot of multivariable logistic regression analysis of factors associated with long-term prognosis in patients with AE

Factors with *p* < 0.05 in the univariate analysis were included in the multivariate logistic regression. Consciousness impairment (OR 3.738, 95%CI 1.069–13.068, *p* = 0.039; OR 4.506, 95%CI 1.137–17.867, *p* = 0.032; OR 4.255, 95%CI 1.056–17.140, *p* = 0.042) and poor short-term prognosis after first-line immunotherapy (OR 14.707, 95%CI 2.865–75.491, *p* = 0.001; OR 6.425, 95%CI 1.108–37.254, *p* = 0.038; OR 8.294, 95%CI 1.464–47.807, *p* = 0.017) were independent risk factors for poor prognosis in patients with anti-NMDAR encephalitis at 6 months, 12 months, and 24 months (Fig. 6).

The treatments administered after the initial immunotherapy included no additional treatments, repeated first-line therapy, second-line therapy, and combined first- and second-line immunotherapy. The selection of treatment episodes is reported in Supplementary Tables 2 and no significant correlation between treatments administered after the initial immunotherapy and prognosis (6 months, 12 months, 24 months, 36 months, and 48months) was identified. The recurrence rate in this cohort was 11.6%. The percentage of patients who relapsed within 12 months was 30.77%, 61.54% relapsed within 24 months, and 76.92% relapsed within 36 months. A total of 15.38% of patients had more than one relapse. The longest interval between recurrence and the first episode was 69 months.

## Discussion

Although previous studies found that central hypoventilation, consciousness impairment, autonomic dysfunction, ICU admission and delay in immunotherapy appeared to associate with poor prognosis in patients with AE, the results of different studies were conflicted [16, 20–22]. This may be due to the heterogeneous of AE. Prognosis is widely variable depending on the subtypes, using those factors to predict the prognosis of one single subtype of AE is imprecise. In this study, we focused on anti-NMDAR encephalitis, the most common type of AE, to construct a more accurate prognostic model. Despite several studies previously identified some prognostic factors predicting short-term prognosis after first-line immunotherapy in patients with anti-NMDAR encephalitis [12, 23, 24], to our knowledge, our study is the first to construct a nomogram. This is a simple-to-use visualization tool for neurologists to predict the short-term prognosis early and adjust treatment timely.

Through multivariable analysis, we identified abnormal behavior or cognitive dysfunction, autonomic dysfunction or central hypoventilation, consciousness impairment, CSF pleocytosis and abnormal EEG as independent prognostic factors for predicting poor short-term prognosis after first-line immunotherapy. The nomogram that incorporated those factors demonstrated good discrimination and sufficient calibration both in derivation cohort and validation cohort.

Abnormal behavior or cognitive dysfunction is a common manifestation of anti-NMDAR encephalitis [11, 25, 26]. It has been reported that cognitive dysfunction may persist for a long time after the acute phase and contribute to long-term morbidity [26, 27]. The same as previous studies [16, 21], we found that abnormal behavior or cognitive dysfunction was linked with worse outcomes. Potentially, this could be attributed to the fact that patients with severe psychiatric symptoms and cognitive dysfunction often require continuous care. Consistent with previous studies [12, 16, 21, 23, 24, 28–30], our research demonstrated that consciousness impairment was a strong predictor of poor prognosis. This could be due to consciousness impairment raising the risk of life-threatening complications such as severe infection, pneumonia and multiple organ dysfunction syndromes. Besides, it was reported that coma was the predominant reason for intensive care unit (ICU) admission [31] and ICU admission was a risk factor for poor prognosis [14, 29]. Autonomic dysfunction or central hypoventilation was also a predictor of poor prognosis, similar to other studies [11, 12, 16, 21–23]. Autonomic dysfunction was another major reason for ICU admission [22]. We hypothesize that it is a trigger factor for ICU-associated complications and further increases the risk of poor prognosis. A recent study demonstrated that paroxysmal sympathetic hyperactivity, which was overlapped with autonomic instability, prolonged neuro-intensive care unit stay, hospital stay and increased duration of mechanical ventilation [32]. Our study found that CSF pleocytosis was associated with poor prognosis, which was supported by fewer previous studies [21, 31]. It could be attributed to the fact that CSF pleocytosis indicates more severe inflammation, which has a negative impact on disease evolution. In addition, previous studies reported that CSF antibody score was associated with CSF white blood cell counts [10], and patients with higher CSF antibody titers tended to be more severe and have more clinical symptoms [8, 28]. Most patients with anti-NMDAR encephalitis have abnormal EEG presentation. The abnormal patterns mainly include diffuse or focal slowing, epileptic discharge, and relatively specific extreme delta brushes [33, 34]. It was reported that > 50% slow waves on EEG were related to poor prognosis [24]. Besides, patients with more severe EEG abnormalities also had significantly longer hospitalized stay and ICU stay [33]. Similar to the previous studies, we found that patients with abnormal EEG tend to have a worse outcome.

This study revealed that the occurrence of combined teratomas in the derivation and validation cohorts was 3.74% and 10.17%, respectively. These percentages were comparatively lower than those reported in previous studies. The dissimilarity in findings may be attributed to the inclusion of a pediatric cohort in our study. Previous research has indicated a lower prevalence of teratomas in pediatric cohorts, particularly among young children. Our observations align with these previous findings [9, 35, 36].

The anti-NMDAR Encephalitis One-Year Functional Status score has already been established and our team has validated it in Chinese patients [18]. This study has several different characters with the previous one. In this study, we did not find any association between abnormal MRI and short-term prognosis. Age distribution and ethnicity are worth considering. The proportion of patients under 18 years (62.7%) was relatively high in our derivation cohort, and it remains unclear whether the impact of abnormal MRI on prognosis differs between children and adults. Besides, previous studies on Caucasian patients suggested that abnormal MRI was associated with poor prognosis [14, 37], whereas studies on Asian patients failed to detect any significant relationship between abnormal MRI and prognosis [21, 38, 39], so we hypothesis that whether abnormal MRI affects prognosis may be related to ethnicity. Inconsistent with previous one, we did not find significant correlations between treatment delay and prognosis, this may because of early diagnosis and appropriate treatment, only 24.6% of patients in our derivation cohort experienced a time to start of treatment after symptom onset longer than four weeks. We did not include ICU stay as a variable because ICU stay may be influenced by medical resources and costs, autonomic dysfunction, central hypoventilation and consciousness impairment reflect ICU requirements more objective to some extent.

The derivation cohort was obtained from a multicenter and prospective clinical registry study of five clinical centers in China. The large sample size and wide geographic distribution of patients in this cohort ensured its representativeness and generalizability for Chinese patients with anti-NMDAR encephalitis. The nomogram included five variables based on clinical manifestations and auxiliary examination findings. Those variables were relatively easy to acquire on admission, making it feasible for application in clinical practice. Our nomogram achieved an AUC of 0.866 with a sensitivity of 0.761 and specificity of 0.869 in the derivation cohort and 0.853 with a sensitivity of 0.720 and specificity of 0.794 in the external cohort, indicating a good performance in distinguishing patients with different prognosis. Additionally, the calibration curve demonstrated good accuracy of the model in predicting prognosis in anti-NMDAR encephalitis patients.

The examination of variables influencing the long-term prognosis revealed that poor short-term prognosis served as an independent risk factor for the long-term prognosis, encompassing prognoses at 6 months, 12 months, and 24 months. Directing attention towards the short-term prognosis facilitated prompt modifications to long-term immunotherapy regimens. Consciousness impairment is a significant predictor of poor short-term and long-term prognoses. This may relate to the fact that patients with consciousness impairment were more likely to require invasive mechanical ventilation, thereby elevating the risk of life-threatening complications, including severe infection, ventilator-associated pneumonia, and multi-organ failure [12, 16, 21, 23, 28–30]. In the analysis of the influence of different treatment regimens on prognosis, no significant correlation between treatments administered after the initial immunotherapy and prognosis (6 months, 12 months, 24 months, 36 months, and 48months) was identified. The reason for these results may be associated with the retrospective nature of this study. The choice of treatment regimens may be affected by the severity of the condition. Patients with more serious illness were more likely to receive maintenance therapy. Therefore, further long-term prospective follow-up studies are warranted to address this issue. As for the recurrence rate, the cohort exhibited a recurrence rate of 11.6% (13 out of 112), aligning with prior findings ranging from 8–25% [40–44]. The relatively low recurrence rate in this cohort may be related to the fact that all enrolled patients received first-line immunotherapy.

The potential limitations of this study should be considered. First, given that patients in this study were all Chinese, we are not sure whether our model is applicable to other populations. This limited the generalizability of our model. Further studies are still needed to validate our findings in other populations. Another limitation is that the information of long-term prognosis was obtained through telephone follow-up, which might have led to some information bias.

## Conclusion

We demonstrated that abnormal behavior or cognitive dysfunction, autonomic dysfunction or central hypoventilation, consciousness impairment, CSF pleocytosis and abnormal EEG as independent prognostic factors for predicting poor short-term prognosis after first-line immunotherapy in patients with anti-NMDAR encephalitis using a multivariable regression model. The nomogram constructed based on those factors could accurately predict prognosis in patients with anti-NMDAR encephalitis. Short-term prognosis is an independent risk factor for long-term prognosis. This practical prognostic model may help neurologists to predict the short-term prognosis early and potentially assist in adjusting appropriate treatment timely.

### Electronic supplementary material

Below is the link to the electronic supplementary material.

Supplementary Material 1

Supplementary Material 2

## Data Availability

The data of the current study are available from the corresponding author on reasonable request.

## Abbreviations

AE: Autoimmune encephalitis
anti-NMDAR: Anti-N-methyl-D-aspartate receptor
AUC: Area under the curve
CI: Confidence intervals
CSF: Cerebrospinal fluid
EEG: Electroencephalogram
ICU: Intensive care unit
IVIG: Intravenous immunoglobulins
MRI: Magnetic resonance imaging
mRS: Modified Rankin scale
OR: Odds ratio
ROC: Receiver-operator characteristic curve
